# Very High Resolution Species Distribution Modeling Based on Remote Sensing Imagery: How to Capture Fine-Grained and Large-Scale Vegetation Ecology With Convolutional Neural Networks?

**DOI:** 10.3389/fpls.2022.839279

**Published:** 2022-05-06

**Authors:** Benjamin Deneu, Alexis Joly, Pierre Bonnet, Maximilien Servajean, François Munoz

**Affiliations:** ^1^Inria, Montpellier, France; ^2^UMR LIRMM, Université de Montpellier, Montpellier, France; ^3^UMR AMAP, Université de Montpellier, Cirad, CNRS, INRAE, IRD, Montpellier, France; ^4^Cirad, Montpellier, France; ^5^Université Paul Valéry, Montpellier, France; ^6^LIPhy, Université Grenoble Alpes, Grenoble, France

**Keywords:** species distribution model, convolutional neural network, ecological interpretation, plant functional traits, t-SNE, very fine scale prediction, remote-sensing imagery

## Abstract

Species Distribution Models (SDMs) are fundamental tools in ecology for predicting the geographic distribution of species based on environmental data. They are also very useful from an application point of view, whether for the implementation of conservation plans for threatened species or for monitoring invasive species. The generalizability and spatial accuracy of an SDM depend very strongly on the type of model used and the environmental data used as explanatory variables. In this article, we study a country-wide species distribution model based on very high resolution (VHR) (1 m) remote sensing images processed by a convolutional neural network. We demonstrate that this model can capture landscape and habitat information at very fine spatial scales while providing overall better predictive performance than conventional models. Moreover, to demonstrate the ecological significance of the model, we propose an original analysis based on the t-distributed Stochastic Neighbor Embedding (t-SNE) dimension reduction technique. It allows visualizing the relation between input data and species traits or environment learned by the model as well as conducting some statistical tests verifying them. We also analyze the spatial mapping of the t-SNE dimensions at both national and local levels, showing the model benefit of automatically learning environmental variation at multiple scales.

## 1. Introduction

Understanding and predicting the spatial distribution of species is a crucial issue in theoretical and applied ecology. In particular, Species Distribution Models (SDMs) are used to characterize the ecological niche of species, i.e., the environmental conditions that explain their presence (Elith and Leathwick, 2009). The ecological niche is inherently multi-dimensional and can involve a large number of factors articulated in a complex manner (i.e., a non-linear system) and at multiple spatial scales. Modern SDMs are generally correlative methods that link known species occurrence data to environmental predictors *via* statistical learning methods (Guisan and Zimmermann, 2000; Guisan and Thuiller, 2005; Peterson, 2011). Among the most popular methods, we can mention MAXENT (Phillips et al., 2006; Phillips and Dudik, 2008) used in a very large number of studies or methods from the machine learning field such as random forest (Cutler et al., 2007) or boosted trees (De'ath, 2007; Elith et al., 2008). The latter generally allow substantial gains in predictive performance, but sometimes at the expense of weaker ecological interpretability. More recently, SDMs based on deep neural networks have emerged to try to better characterize the high complexity of how the environment shapes ecological niches (Chen et al., 2016; Benkendorf and Hawkins, 2020). These deep learning models have several advantages: (i) they can efficiently capture complex relationships from a very large number of predictors, (ii) they can extract ecological features common to a large number of species and thus capture fundamental ecological patterns, and (iii) they can significantly improve the prediction quality of species compositions (Botella et al., 2018; Christin et al., 2019).

A particular type of neural network initially proposed by LeCun et al. (1989), named convolutional neural networks (CNN), has recently been introduced for the modeling of species distribution (Botella et al., 2018; Deneu et al., 2018; Gillespie and Exposito-Alonso, 2020). The main added value of these CNN-SDMs models compared to non-convolutive deep neural networks and other machine learning methods is that they are based on spatial environmental tensors rather than on point values of environmental variables. These tensors capture the spatial dimension of the environmental variables around each point in addition to their value. Unlike classical SDMs, the great strength of CNN-SDMs is to be able to extract relevant spatial-environmental patterns from such complex input data (Deneu et al., 2021). CNNs were originally designed for image classification, inspired by convolution operators used in signal processing. For a long time, their use remained limited because their training requires significant hardware resources and large volumes of training data. For nearly 10 years, they have been recognized as undeniably more efficient than any other method for tasks requiring the extraction of information from images (especially multi-channel images). Therefore, within a few years, CNNs have become increasingly popular in the field of ecology for various tasks such as identifying species, classifying animal behavior, or estimating biodiversity in camera-trap images, videos, and audio recordings (Christin et al., 2019).

In this article, we study an SDM based on a convolutional neural network trained with very high resolution (VHR, 1 m) remote sensing images as one of the input variables. Its fitting on millions of plant and animal occurrences (coming from citizen science) required several weeks of computation on a GPU-equipped super-computer. The evaluation of its performance on several thousand species shows that it is superior to a state-of-the-art environmental SDM while its spatial resolution is several orders of magnitude higher. Beyond the raw predictive performance, we then focus on the ecological interpretation of this unusual SDM. Therefore, we employed a t-SNE (Maaten and Hinton, 2008), a non-linear dimension reduction method widely used to visualize the feature space learned by deep learning models. Specifically, we use t-SNE to construct a low-dimensional (2-dimensional and 3-dimensional) embedding of the high-dimensional representation space learned by the CNN (i.e., of the 2,048-dimensional feature vectors used as the input of the final species classifier). These low-dimensional representations are then exploited in three ways: (i) to visualize in geographic map form the spatial patterns of habitats and landscapes learned by the model, (ii) to visualize in graphical form the relationships between learned features, environment, and species traits, and (iii) to quantitatively verify these relationships using statistical tests. This interpretability study demonstrates that our CNN-SDM trained on VHR remote sensing data captures the landscape and habitat information at fine spatial scales while providing better overall predictive performance than conventional models. This offers the possibility to produce large-scale distribution maps for a large number of species simultaneously and at a spatial resolution rarely equalled. Moreover, it opens the possibility to analyze their consistency with the fine ecological knowledge of each species, which is almost impossible with coarser approaches. The conducted statistical tests also clearly demonstrate that the model is able to capture meaningful environmental and ecological patterns from the input data. This is particularly remarkable in the sense that none of these data were used as input variables during the training of the model. In addition, statistical tests show that the features learned by the model are significantly related to the environment and species traits. The model is able to extract this high-level information directly from the raw data used as input (the spatial-environmental tensors coupled to species occurrences).

## 2. Materials and Methods

### 2.1. CNN-SDM Model Training and Validation

#### 2.1.1. Training Dataset

**Figure 1:**
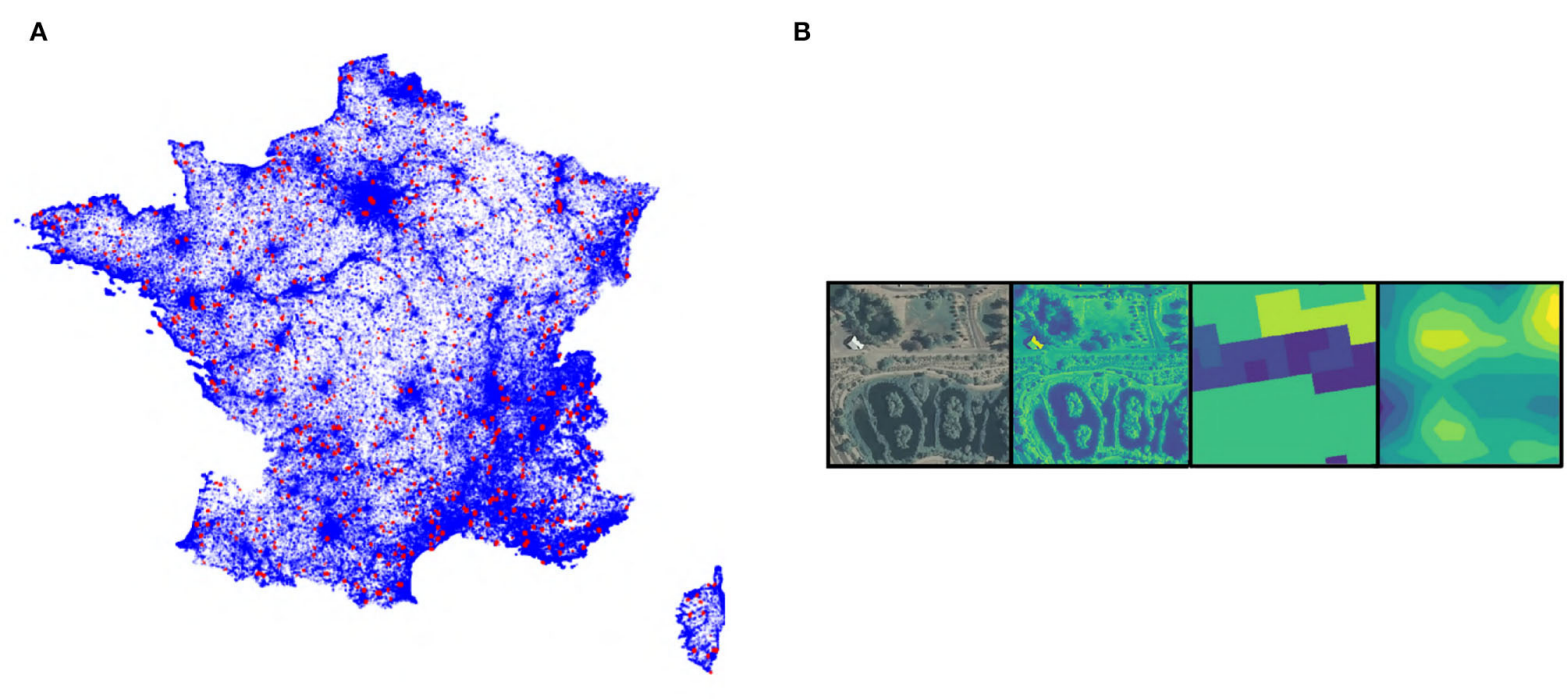
**(A)** Observations distribution (training data in blue, test data in red) in France. **(B)** Example of a very high-resolution (VHR) tensor of 256 × 256 m square with, respectively, RGB images (native colors), Near-IR images (artificial colors), land cover (artificial colors), and altitude (artificial colors). Artificial colors are from purple (lowest values) to yellow (highest values).

For this study, we use the GeoLifeCLEF 2020 dataset, a detailed description of which is provided in Cole et al. (2020). This dataset covering France and the USA consists of 1,921,123 observations (8,23,483 in France and 1,097,640 in the USA) belonging to 31,435 different species, mainly plants and animals. Each observation is coupled to a tensor extracted from remote-sensing data (at high or VHR) at the position of the occurrence, refer to Figure 1. The four remote-sensing data used are, RGB and Near-IR imagery (from the 2009-2011 cycle of the National Agriculture Imagery Program (NAIP) in the United States^1^, and the BD-ORTHO® 2.0 and ORTHO-HR® 1.0 from IGN^2^ in France), land-cover (National Land Cover Database (NLCD) (Homer et al., 2015) for the United States and CESBIO^3^ for France), and elevation (Shuttle Radar Topography Mission (SRTM)^4^ for both France and USA). Table 1 summarizes the data sources and native resolution. These different remote-sensing data have been standardized to a spatial scale of 1 m per pixel. The finest data were downsampled (including for example the ORTHO-HR data which was up to 20 cm resolution) and the elevation and land-cover were oversampled. The oversampling of the elevation data is done using a bilinear interpolation that smoothes the interpolated data to avoid sharp edges to which the CNN can be sensitive (note that the data provided is itself already interpolated). On the contrary, the oversampling of categorical land cover data is done without interpolation for obvious reasons of data degradation (only the nearest neighbor allows us to keep the classes intact). The final tensors are 256 × 256 pixels covering 256 × 256 m for each data and centered on the position of each observation. An example is given in Figure 1B.

**Table 1:** Summary of data sources.

**Name**	**Description**	**Native resolution**
NAIP	RGB and Near-IR imagery (US)	1 m
BD-ORTHO® 2.0	RGB and Near-IR imagery (France)	0.5 m
ORTHO-HR® 1.0	RGB and Near-IR imagery (France)	0.2 m
NLCD	Land-cover (US)	30 m
CESBIO	Land-cover (France)	10 m
SRTM	Elevation	1 arcsec (≈30 m at equator)

#### 2.1.2. Deep Convolutional SDM Architecture

Our deep convolutional neural network is the composition of non-linear transformations (including the convolutional layers) of the input space ***z*** = ϕ(***x***) with a linear classifier ψ(***z***) which is trained in a similar way to a multinomial logistic regression, i.e., by minimizing the negative log likelihood of:


(1) $$\mathbb{P}(Y=k⏐X=x)=\sigma_{k}(\psi (z))=\frac{e^{\psi_{k}(z)}}{\sum_{j}e^{\psi_{j}(z)}}$$

where σ is the softmax function that maps the logit ψ_*k*_(***z***) of a particular species to its relative probability. The vector ***z*** is called the feature vector (or representation vector) of the input tensor ***x***. Here, the size of the feature space is 2,048, it is defined by the architecture of the model. We use the Inception V3 (Szegedy et al., 2016) model architecture adapted in the same way introduced in Deneu et al. (2020b) to fit the format of the input data and the number of output classes (species). The model is trained using the cross-entropy loss and so the outputs of the model can be interpreted as relative probabilities of occurrence of species for input data ***x***.

#### 2.1.3. Environmental and Trait Data

In this study, we also use environmental and species trait data. These data are not used for training the CNN but are used to study the ability of the model learned on VHR data to extract information related to the ecology of species. We use 19 bio-climatic rasters [30 arcsec^2^/pixel (above 1 km^2^/pixel) from WorldClim (Hijmans et al., 2005)] and 8 pedologic rasters [250 m^2^/pixel, from SoilGrids (Hengl et al., 2017)]. The detailed list and resolutions are presented in Table 2. This environmental data is also used to train an environmental random forest in order to compare this more classical approach and its performance to our model (refer to Section 2.1.4).

**Table 2:** Summary of environmental rasters.

**Name**	**Description**	**Res**.
bio_1	Annual Mean Temperature	30 arcsec
bio_2	Mean Diurnal Range [Mean of monthly (max temp - min temp)]	30 arcsec
bio_3	Isothermality (bio_2/bio_7) (* 100)	30 arcsec
bio_4	Temperature Seasonality (standard deviation *100)	30 arcsec
bio_5	Max Temperature of Warmest Month	30 arcsec
bio_6	Min Temperature of Coldest Month	30 arcsec
bio_7	Temperature Annual Range (bio_5-bio_6)	30 arcsec
bio_8	Mean Temperature of Wettest Quarter	30 arcsec
bio_9	Mean Temperature of Driest Quarter	30 arcsec
bio_10	Mean Temperature of Warmest Quarter	30 arcsec
bio_11	Mean Temperature of Coldest Quarter	30 arcsec
bio_12	Annual Precipitation	30 arcsec
bio_13	Precipitation of Wettest Month	30 arcsec
bio_14	Precipitation of Driest Month	30 arcsec
bio_15	Precipitation Seasonality (Coefficient of Variation)	30 arcsec
bio_16	Precipitation of Wettest Quarter	30 arcsec
bio_17	Precipitation of Driest Quarter	30 arcsec
bio_18	Precipitation of Warmest Quarter	30 arcsec
bio_19	Precipitation of Coldest Quarter	30 arcsec
bdticm	Absolute depth to bedrock in cm	250 m
bldfie	Bulk density in kg/m3 at 15 cm depth	250 m
cecsol	Cation exchange capacity of soil in cmolc/kg 15 cm depth	250 m
clyppt	Clay (0-2 micro meter) mass fraction at 15 cm depth	250 m
orcdrc	Soil organic carbon content (g/kg at 15 cm depth)	250 m
phihox	Ph x 10 in H20 (at 15 cm depth)	250 m
sltppt	Silt mass fraction at 15 cm depth	250 m
sndppt	Sand mass fraction at 15 cm depth	250 m

We also use data related to the ecology of the species, more precisely Ellenberg indicator values (EIVs, refer to Table 3) (Ellenberg, 1988) from Julve (1998). These data are available for more than 1,400 plant species that we have in our dataset. These variables consist of an ordinal classification of ecological strategies with respect to major environmental constraints and essential resource use (Bartelheimer and Poschlod, 2016).

**Table 3:** Summary of Ellenberg's plant species traits data.

**Name**	**Description**	**Ranges of values (Nb species)**
EIV L	Light availability	2–9 (1,423)
EIV T	Temperature	1–9 (1,413)
EIV K	Climatic continentality	1–8 (1,411))
EIV AirH	Air humidity	1–9 (1,405)
EIV F	Soil humidity	1–12 (1,405)
EIV R	Reaction (soil acidity / pH)	1–9 (1,410)
EIV TroL	Trophic level	1–9 (1,412)
EIV S	Salt (soil salinity)	0–9 (1,416)
EIV SoiT	Soil texture	1–9 (1,416)
EIV N	Nitrogen (soil fertility)	1–9 (1,422)

#### 2.1.4. Environmental Random Forest

For performance comparison purposes, we also train an environmental random forest model. This more classical model is trained using environmental rasters of climate and soil data (refer to Section 2.1.3). We extract the environmental realization (the value taken by the environmental variables) at the point of each occurrence, which associates an environmental vector of size 27 with each occurrence. Similar to CNN, the random forest is learned on all the training occurrences over France and the USA, with the exception of some occurrences in Florida which are out of the coverage of the provided raster and are removed (there is no such problem on the test occurrences). We use the random forest classifier of scikit-learn (Pedregosa et al., 2011). The optimization of the parameters is done using a validation set of occurrences made of 0.5% of the occurrences randomly extracted from the training set. The final parameterization consists of a forest of 100 trees with a maximum depth of 10, the other parameters are left at their default values. The predictive power of random forests generally increases with the number of trees and the depth. However, the memory size of the model increases exponentially with the depth. Due to the size of the dataset, a depth of 10 was a limit with the resources we had available and using the scikit-learn implementation.

#### 2.1.5. Models Evaluation

Both models (CNN and random forest) were evaluated in the context of the GeoLifeCLEF 2020 challenge (Deneu et al., 2020a; Joly et al., 2020). The main metric used for evaluation is the *top-k accuracy* index, i.e., the probability that the true species of the observation belongs to the set of *k* species predicted as most likely by the model. This metric is well-adapted to classification models that return relative probabilities such as the CNN or the random forest classifier. It also has the advantage of not requiring absence or pseudo-absence data. For an observation i∈D, we defined as *r*_*i*_ the rank of the true species of *i* in the sorted list of the estimated probabilities ŷ_*s*_(*i*). Additionally, for *k*≥1, we defined the *top-k accuracy* as:


(2) $$A_{k}=\frac{\sum_{i}^{n}A_{k}(i)}{n}$$

with *n* is the number of occurrences in the test set and


(3) $$A_{k}(i)=\left\{ \begin{array}{l} 1\\ 0 \end{array}\right.\;\begin{array}{l} \text{if}\${\text{r}}_{\text{i}}\$\leq \text{k}\\ \text{else} \end{array}$$

To avoid giving too much weight to the most frequent species, it is preferable to evaluate the models in terms of scores per species and not per occurrence. Therefore, we defined the *species-wise top-k accuracy* for a particular species *s* as:


(4) $$SA_{k,s}=\frac{\sum_{i}^{j}A_{k}(i)}{j}$$

With *j* the number of occurrences of species *s* in the test set. Then we defined the *mean top-k accuracy* per species by:


(5) $$\begin{array}{c} MSA_{k}=\frac{\sum_{s}^{n}SA_{k,s}(m)}{n} \end{array}$$

with *n* the number of species in the test set. For the comparison of the two models, we choose to set *k* to 30. Models are evaluated using a spatial block holdout procedure (i.e., test occurrences are contained in 5 × 5 km quadrats with no train occurrences and represent 2.5% of the overall set).

In addition, we also evaluate the models with the Area Under Curve metric (AUC), more commonly used by the ecology community. For that, it is necessary to establish a method for selecting pseudo-absences. As the data we use are provided only at the points of occurrences, the pseudo-absences of a species must be drawn among the occurrences of other species. A naive draw of pseudo-absences could then be simply to draw randomly in all other occurrences uniformly. However, the distribution of occurrences by species as well as spatially being highly imbalanced could introduce biases in the representativeness of habitats in the pseudo-absences. To address this problem we propose to balance the drawing of pseudo-absences on the species and not the occurrences. Each pseudo-absence is then chosen by first randomly drawing a species (other than the one evaluated and with at least 1 occurrence in the test set) and then by randomly drawing a test occurrence of this species. For each species, we draw at least 100 pseudo-absences or as many as presence if the species has more than 100 occurrences in the test set. The AUC is then computed by species and the models are compared by their average AUC by species (*MeanAUC*). Note that the outputs of the random forest are the relative probabilities of the species. It is the score that is used to compute the AUC. However, for the neural network, it is the activation of the last linear layer (prior to the softmax, i.e., the logits of the species) that is used because it is closer to habitat suitability. Contrary to *MSA*_*k*_ and *A*_*k*_, the AUC is dependent on the pseudo-absences and their “environmental” distance from the presences, so the extent of the study area greatly influences the score obtained. The models being learned on a particularly large geographical area and covering the two countries, France and US, we also propose to evaluate the *MeanAUC* for each country separately.

### 2.2. Dimension Reduction

The learning of CNNs is done through a representation space, also called feature space. This space is concretized as the last layer of the CNN on which a linear model gives the final output. Each occurrence gives an activation of the neurons of this layer noted **z** = ϕ(**x**). This space then concentrates the information captured by the model in the input data in such a way that the species tend to be linearly separable. Analysis of the structure of this feature space and the information it has captured can lead to a better understanding of the explanatory factors captured by the model and how they relate to the ecology of the species. However, the feature space remains a relatively high-dimensional space (2,048) which makes it difficult to perform both qualitative and quantitative analyses on the space as is. We, therefore, propose to project this space and thus the vectors of activations (**z**) of the occurrences in a new space of very low dimension (2 or 3 dimensions). For this, we use a state-of-the-art non-linear dimension reduction method, the t-SNE (Maaten and Hinton, 2008). The t-SNE algorithm is based on a probabilistic interpretation of proximities. A probability distribution is defined for the pairs of points in the source space such that points that are close to each other have a high probability of being selected while points that are far away have a low probability of being selected. A probability distribution is also defined in the same way for the visualization space. The t-SNE algorithm consists of matching the two probability densities, minimizing the Kullback-Leibler divergence between the two distributions with respect to the location of points on the map. The main advantage of t-SNE is that it is able to preserve the local structure of the original space even if it is not linear. Since t-SNEs aim to preserve local similarities, a common practice is to perform a first dimension reduction before t-SNEs for very large dimensionalities. This helps to preserve the global structure as well and to improve the computation time. Here, we choose to use a PCA as a preliminary dimension reduction step (Kobak and Berens, 2019).

Specifically, to process the dimension reduction, we randomly selected 32, 000 training occurrences **x**^*j*^ and computed their representations **z**^*j*^ = ϕ(**x**^*j*^). Then, we first reduced the dimension from 2,048 to 50 by PCA using the scikitlearn package. The resulting 50-dimensional feature vectors were then further reduced by t-SNE, also using the scikitlearn package. For most experiments, the used dimension for t-SNE was set to 2 (apart from the geographical map of **Figure 6** where it was set to 3 without PCA and based on other occurrences, refer to Section 2.4). In the following, we denote as $\tilde{\text{z}}=g\left(\text{z}\right)$ the resulting 2-dimensional feature vectors, where the function *g* denotes the complete dimension reduction function (PCA+t-SNE).

### 2.3. Learned Space Visualization

We propose to illustrate the main information related to the landscape, the environment, or the ecology of the species captured by the model using several visualizations on the two-dimensional t-SNE space. To do so, we discretize the two-dimensional space as a grid of size *n* × *n* with *n* the number of discretizations that can be arbitrarily chosen. On this grid, we search in each cell with at least one occurrence for the occurrence that is closest to the center of the cell (knn function in scikitlearn). Thus, we associate with each cell an occurrence (if there is one). We can then produce different visualizations by displaying information or data related to the occurrences at their position in the grid. Each cell, which can then be seen as a pixel, displays the data of its associated occurrence. The first one (**Figure 3**), consists in displaying the RGB image corresponding to the input data of the occurrence (which is equivalent to taking the tensor associated with the occurrence but keeping only the R,G,B channels, and not the Near-IR, Altitude, and land-cover channels). The other visualizations (**Figure 8**) display the environmental realization at the point of the occurrence or the value of a trait of the species of this occurrence.

The second representation is a projection of the t-SNE space on the geographical space. To do this, we first applied a bilinear color gradient on the t-SNE space (refer to **Figure 5A**), we then took a grid of points covering the French territory with one point for each kilometer. Each geographical point was associated by a 1-Nearest Neighbor algorithm to the closest occurrences used in t-SNE. The point then took the color obtained in t-SNE space for the corresponding occurrence and provided a pixel of the map in **Figure 5B**.

### 2.4. Very High Spatial Resolution Analysis

In addition to the visualization of learning on the whole territory, we demonstrate a visualization at a finer scale and VHR. To do so, we analyzed the activation of the model in a selected geographical area. The choice of the area was made according to two criteria. (1) The area had to contain several distinct environments within a small spatial extent (about 10 km). (2) The area had to be sufficiently documented and known by the authors to draw relevant conclusions. The result is the choice of an area of 10 × 15 km on the French Mediterranean coast close to Montpellier city, including a massif, coast line, salt ponds, garrigues, pine forests, agricultural areas, vineyards, urban areas, and some significant human infrastructure such as a highway. The model activation was performed every 50 m after extracting the input data for these points following the same procedure as for the official data set described in Cole et al. (2020). We then performed two different visualizations on this area. The first one is a visualization of the feature space **z** *via* a dimension reduction by t-SNE similar to what is described in Section 2.2 with some differences. Unlike the t-SNE described, here, we did not apply a PCA and we performed a reduction to three dimensions instead of two. We then plotted the 3-dimensional t-SNE on the map by re-scaling each of the three axes such as the values felt within 0–255. Each point could then be associated with an RGB color from its coordinates on t-SNE space. The resulting map was the plot of each point as a color pixel at its geographical position. The second representation was a geographical display of the activation of the logit of four species in this area (the logit being the confidence score associated with each species as presented in Equation 1). We suppose here that the logit provides an index of habitat suitability. We chose the species to be representative of different environmental conditions at a fine scale. The four selected species were *Ailanthus altissima* (Mill.) Swingle. (invasive along roads, rails, etc.), *Cistus albidus* L. (in the garrigues and limestone slopes), *Capsella bursa-pastoris* (L.) Medik. (pioneer of lawns, crops, wasteland, rubble in urban areas), and *Anthemis maritima* L. (marine sands).

For this analysis, with a limited number of species, we propose to compare our model to the predictions of an environmental *MaxEnt* (Phillips et al., 2006; Phillips and Dudik, 2008) model for the 4 species mentioned. The *MaxEnt* model remains to this day the most used model for single species SDMs and is known for its performance. We used the R implementation of *MaxEnt* and the environmental rasters presented in Section 2.1.3 for the 4 species models. We give as input to *MaxEnt* the bioclimatic and soil rasters over France. As the rasters must be given with the same size and resolution we use the “resample” function of the “aster” library in R to scale the bioclimatic raster to those of the soil (250 m). For the species *Anthemis maritima* L., being a coastal sand species, more than half of the learning occurrences fell outside the coverage of the rasters (the rasters being defined only on land, the occurrences too close to the water can be in the no data cells). To overcome this problem, we use the “approxNA” function of the “raster” library which allows us to extend the coverage of the rasters by replicating the values close to the no data zones onto them. The prediction of the *MaxEnt* models in this area can be compared to the CNN logit activations to see the difference in dynamics and resolution of the two models. However, there is a conceptual difference between the outputs of the models. Where *MaxEnt* gives an estimate of the probability of presence, the CNN logits cannot be interpreted as such. The two predictions are therefore not directly comparable. We propose only to compare the spatial dynamics of the maps produced in this area and not to directly compare the presence/absence predictions. To do so, we scale, for each species, the two model outputs to a prediction between 0 and 1, where 1 corresponds to the point on the map where the score given to the species by the model is the highest and 0 the lowest. This method is justified by the choice of species that we know are present in the study area but not in all environments. In other words, we know that these species are present in some habitats included in the study area and absent from some others. In practice, the output of the *MaxEnt* is already between 0 and 1 so we simply use a min-max scaler to set the local maximum to 1 and the local minimum to 0. For the CNN, the logits are not necessarily between 0 and 1 and can contain extreme values, thus, we first apply a sigmoid to bring the logits scores between 0 and 1 and then the min-max scaler. The threshold of the sigmoid is chosen equal to the average activation of the logits of the species in the area. This allows to center the values and remains consistent with the known condition of absence and presence of the species in the area.

We rendered the maps by combining a background map of the remote sensing data and a layer displaying the values obtained with a colormap going from transparent (0) to bright red (1).

### 2.5. Ecological Interpretation of the Learned Features

Here, we use the species, ecological traits and environment data that were not used during model training (refer to Section 2.1.3). These data allow us to assess how well the model is able to capture information related to the environment (climate and soil factors) and species ecology (species traits). To do so, we fit a linear model on each axis of the t-SNE (the two variables ${\tilde{\text{z}}}_{1}$ and ${\tilde{\text{z}}}_{2}$) using either the environmental or ecological trait variables as explanatory variables (R language, lm function). The correlation then captured between the feature space of the model (reduced by t-SNE) and these data may be indicative of the ability of the CNN model to capture information directly related to species ecology through VHR imagery data. The variable bio_7 was removed from the linear model on the environmental variables because by definition it is equal to bio_5−bio_6 and is, therefore, directly correlated to them.

## 3. Results and Discussion

**Figure 2:**
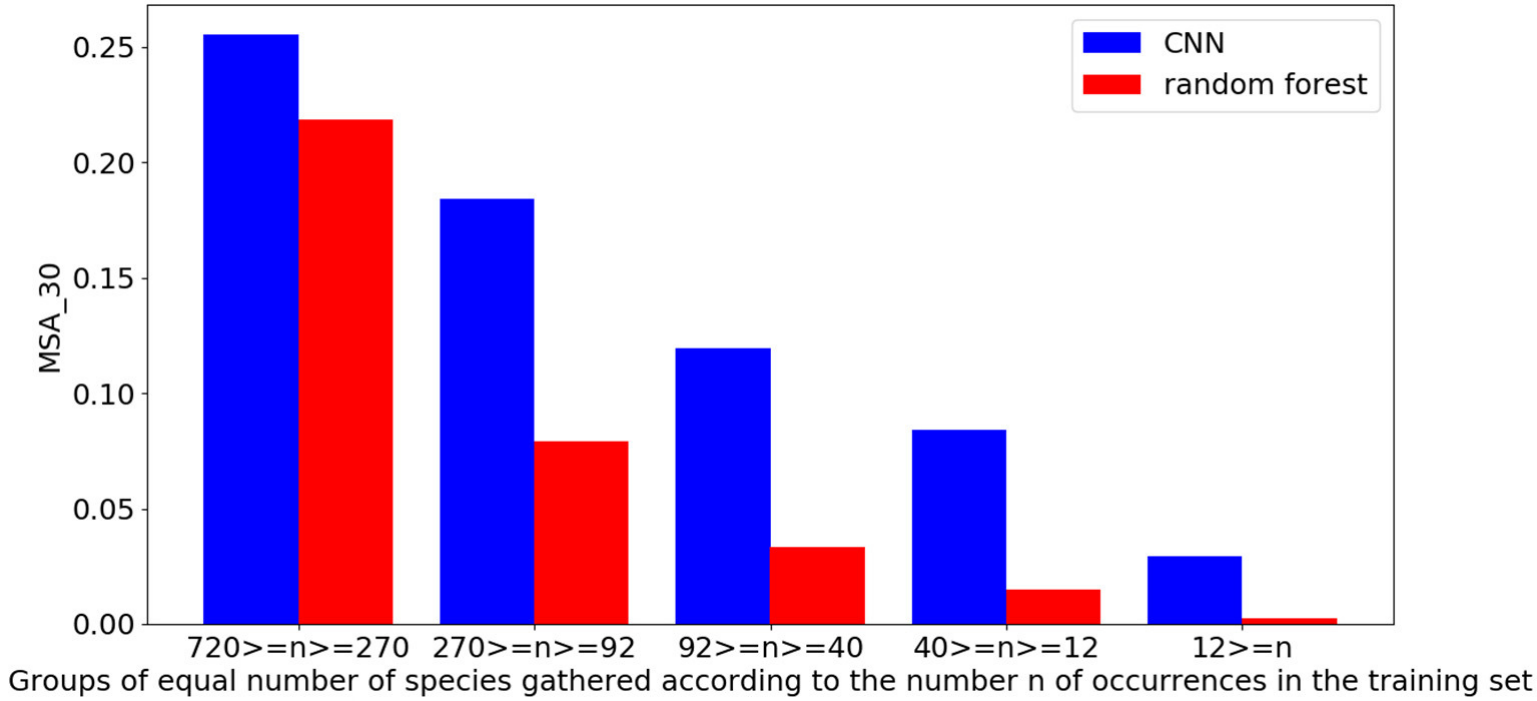
Performance of models in mean top-30 accuracy per species (*MSA*_30_) according to the frequency of the species in the training set.

The evaluation of the CNN based on VHR remote-sensing data against the environmental random forest highlights the performance of the CNN which obtains a better score than the more classically used environmental model on all metrics (Table 4). The CNN obtains 23.5 vs. 20.4% on the *top-30 accuracy* (*A*_30_). The performance gap is even greater when evaluating the *mean top-30 accuracy* per species (*MSA*_30_) with a score of 13.2% for the CNN against 6.9% for the random forest. This suggests that the CNN is particularly better on less represented species in the dataset as these species gain more weight in the *MSA*_30_ compared to the *A*_30_. Figure 2 confirms this by showing the performance of the two models as a function of the number of occurrences in the training set. The difference in the performance of the two models increases rapidly as the number of occurrences decreases. In particular, for species between 270 and 92 occurrences, the CNN is already twice as good as random forest.

**Table 4:** Evaluation of the models.

**Model**	***A*** **_30_**	***MSA*** **_30_**	***MeanAUC***	***MeanAUC*** **France**	***MeanAUC*** **US**
Environmental random forest	0.204	0.069	0.905	0.732	0.889
Remote-sensing based CNN	0.235	0.132	0.915	0.771	0.902

The model evaluation in *MeanAUC* shows a good overall capture of the species distribution over the global dataset with both models over 0.9 (0.915 for the CNN and 0.905 for the random forest). However, the separate *MeanAUC* evaluation shows lower scores than the overall evaluation for both countries. This illustrates the impact of pseudo-absence on particularly large and diverse study areas. In particular, the scores in France are significantly lower than in the US or the global evaluation. Two factors may be at play, the task may be more difficult (more species with few occurrences) but also the smaller size of the country may accentuate the previous remark. The comparison of the models confirms with this metric the better performance of the CNN against the random forest. In particular, in France, where the difference of *MeanAUC* is the most important (0.771 for the CNN vs. 0.732 for the random forest) shows that the CNN seems more robust when the task is more difficult.

Both proposed metrics have limitations that must be taken into account in the analysis of the results. For the evaluation of *MSA*_*k*_ or *A*_*k*_, the most obvious problem is the choice of *k*. Indeed, the actual *k* depends on both the spatial resolution of the prediction and the specific richness at the prediction point, which we do not know. The relative probability given to a rare species by the model may never allow it to reach the top *k* for small *k* even if the spatial dynamics of its prediction are consistent with its distribution. In our case, the choice of a relatively restrictive k with respect to the number of species (k fixed at 30 for 31,435 species) leads to relatively low scores in accuracy which can give the impression that the models perform poorly. For the AUC, the choice of pseudo-absences is known to have a strong impact on the score obtained and in particular, in our case, the evaluation of the models in such a large and varied region (France and the United States) facilitates obtaining high AUC scores. Indeed, the drawing of pseudo-absences has a great chance to represent environments that are on average quite far from the species' niches. This partly explains why the evaluations of the two separate countries are lower than the overall evaluation. In general, we prefer to use the *MSA*_*k*_ metric which avoids the choice of pseudo-absences and is adapted to the evaluation of a model learned by cross entropy.

**Figure 3:**
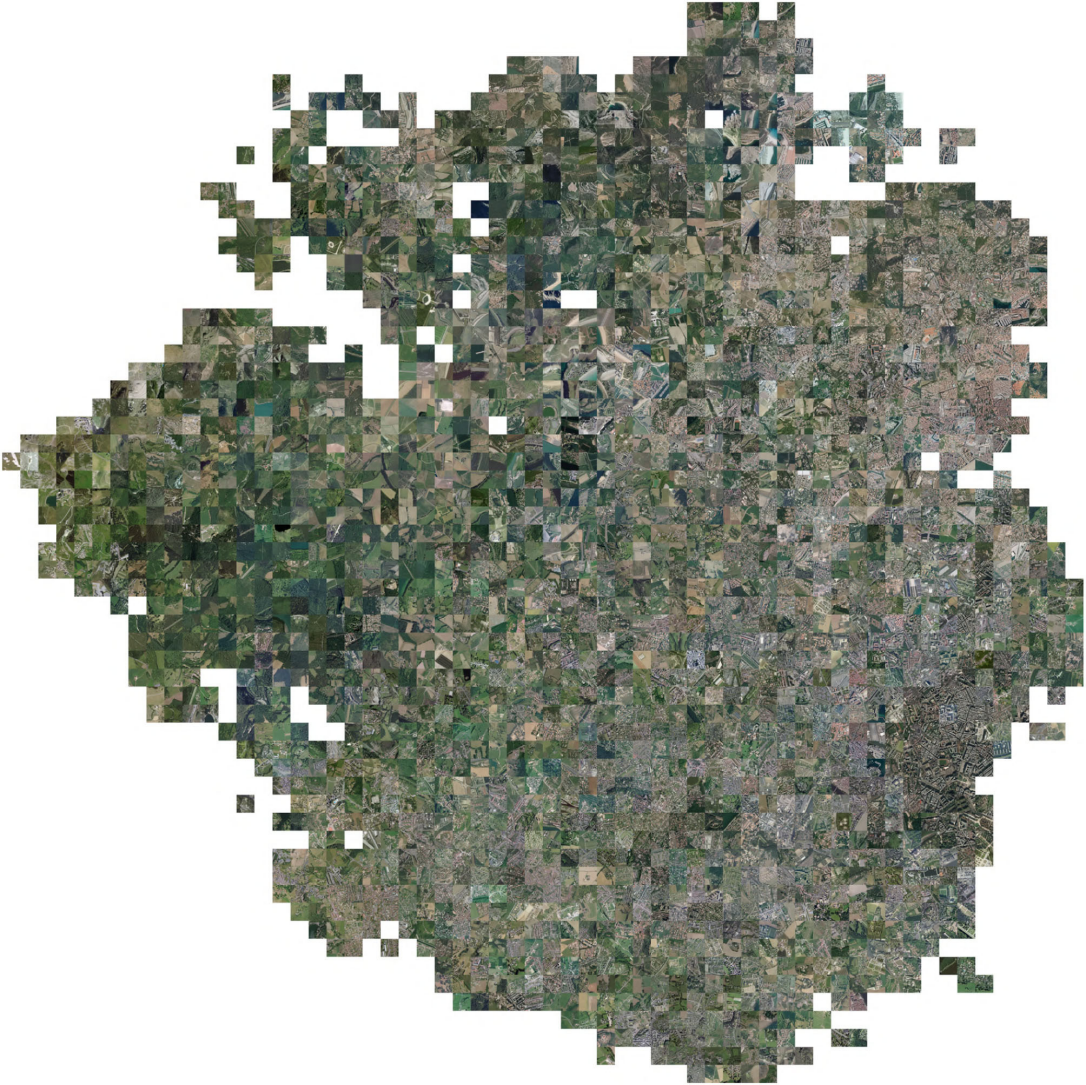
Visualization of the remote sensing imagery patch (RGB) of occurrences on the t-SNE space. The grid corresponds to a uniform slicing of the t-SNE space (the result of the convolutional neural networks (CNN) feature space dimension reduction) and each cell displays the RGB image of the occurrence closest to its center. The position of the occurrences in this space is the result of the dimension reduction of their activation of the feature space.

**Figure 4:**
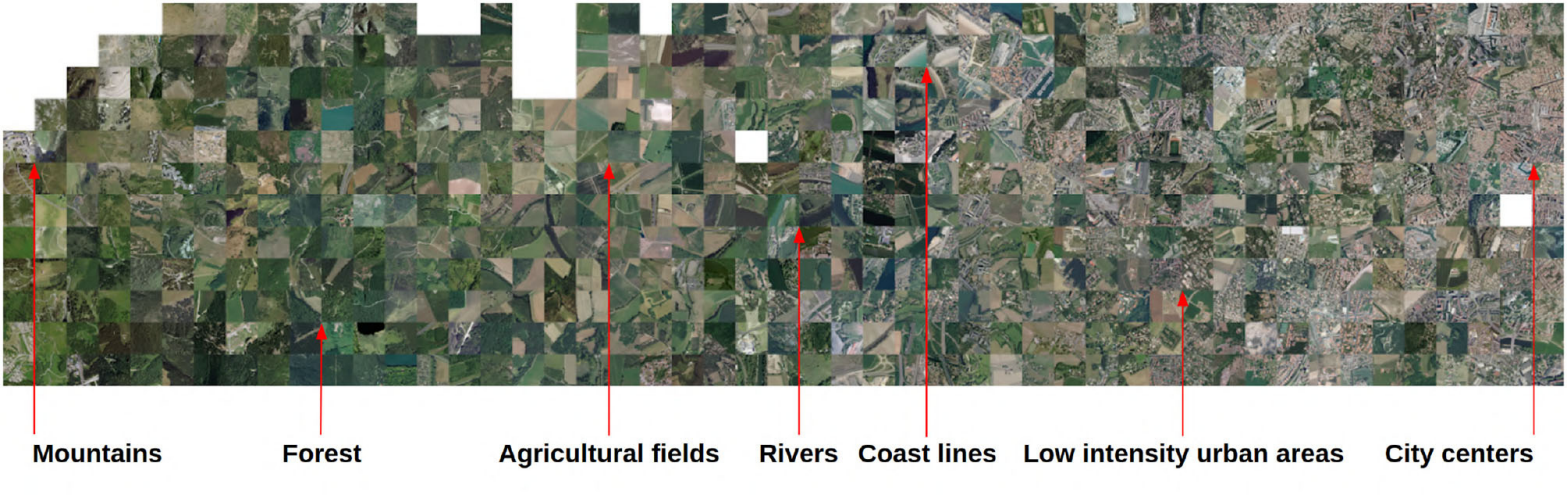
Zoom of Figure 3 with examples of landscape features identified.

**Figure 5:**
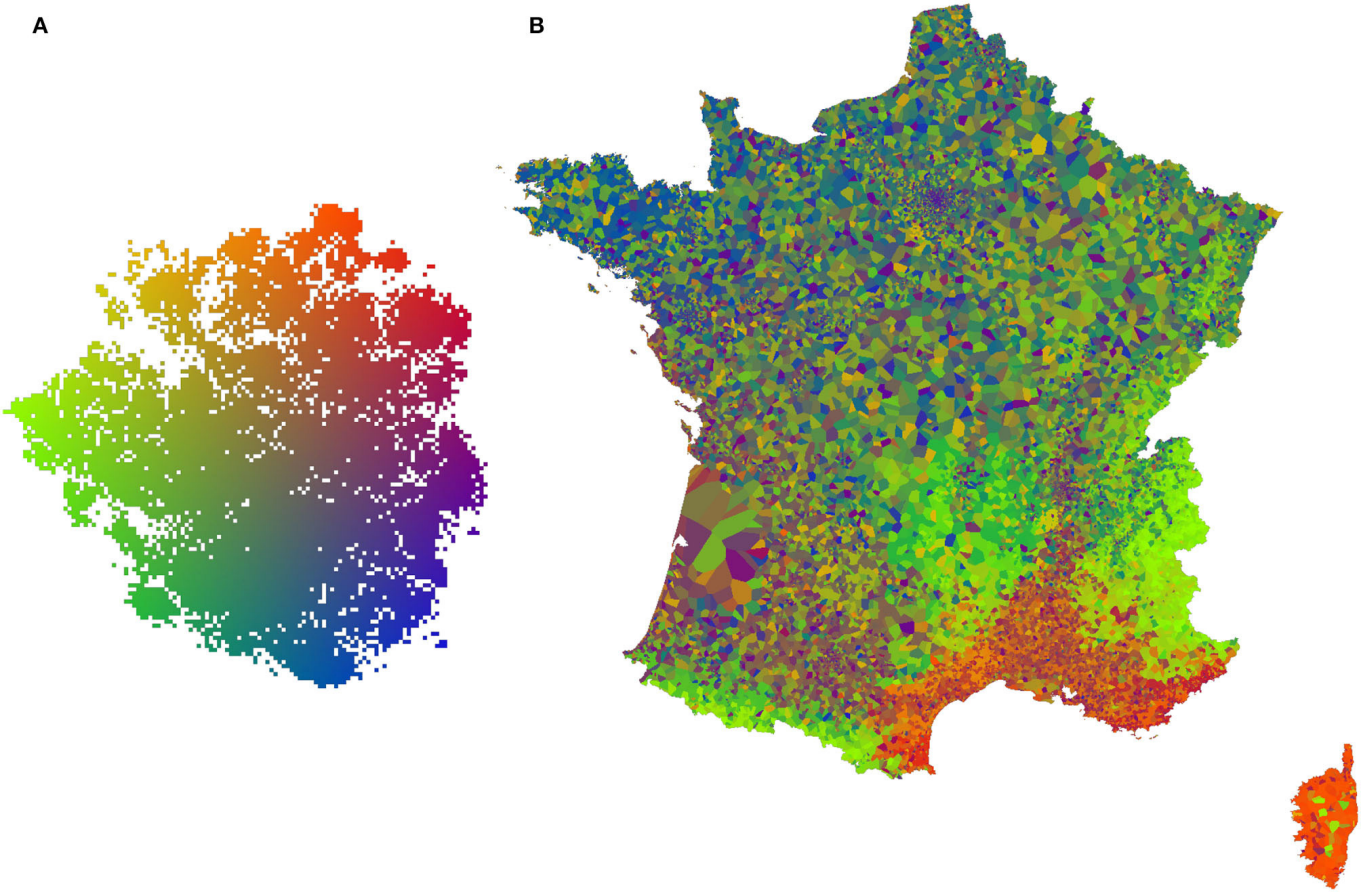
**(A)** Color space applied to t-SNE space (the result of the CNN feature space dimension reduction), each occurrence is associated with a color corresponding to its position in t-SNE space. **(B)** Map of France at 1 km/px resolution, each pixel takes the color of the closest spatial occurrence, a color defined by the **(A)** color space.

Figures 3, 4 visualize the RGB patches of the occurrences on the t-SNE space, and Figure 5 visualizes the projection of the t-SNE space on the geographical space in France. Two complementary conclusions can be drawn from these visualizations. First, the visualization of the RGB patches on the t-SNE highlights the fine-scale landscape factors identified by the CNN. In Figure 3, we can identify several areas of the t-SNE space corresponding to different broad landscape types. On the left side of the t-SNE, we can, e.g., identify mountain patches, and below, we can see forests. Toward the center of the t-SNE, one can identify predominantly agricultural landscapes, and the whole lower part and the right side present more or less dense urban landscapes. Figure 4 presents a zoom on a slice of the previous figure with examples of identifiable landscapes. Second, Figure 5 displays the projection of t-SNE space onto geographic space. It can be seen that the t-SNE space also contains geographic structuring in the large ecoregions. For example, the mountainous areas (e.g., the Alps in the southeast, the Massif Central in the center, and the Pyrenees in the southwest) and the Mediterranean basin stand out in similar colors indicating that these large areas are well-recognized by the model. Overall, the map shows good visual consistency with the large-scale biogeographic zones previously identified (Cervellini et al., 2020). This result combined with the analysis of the previous figure highlights that the model identifies both the broad biogeographic regions but also the different fine-scale landscapes within these regions (such as urban-rural). This important result shows that the model is able to capture spatially consistent information at multiple scales from VHR imagery data with both high spatial accuracy and large-scale consistency. This provides a breakthrough in the well-known problem of trading precision for generality when studying ecological processes (Levins, 1966).

**Figure 6:**
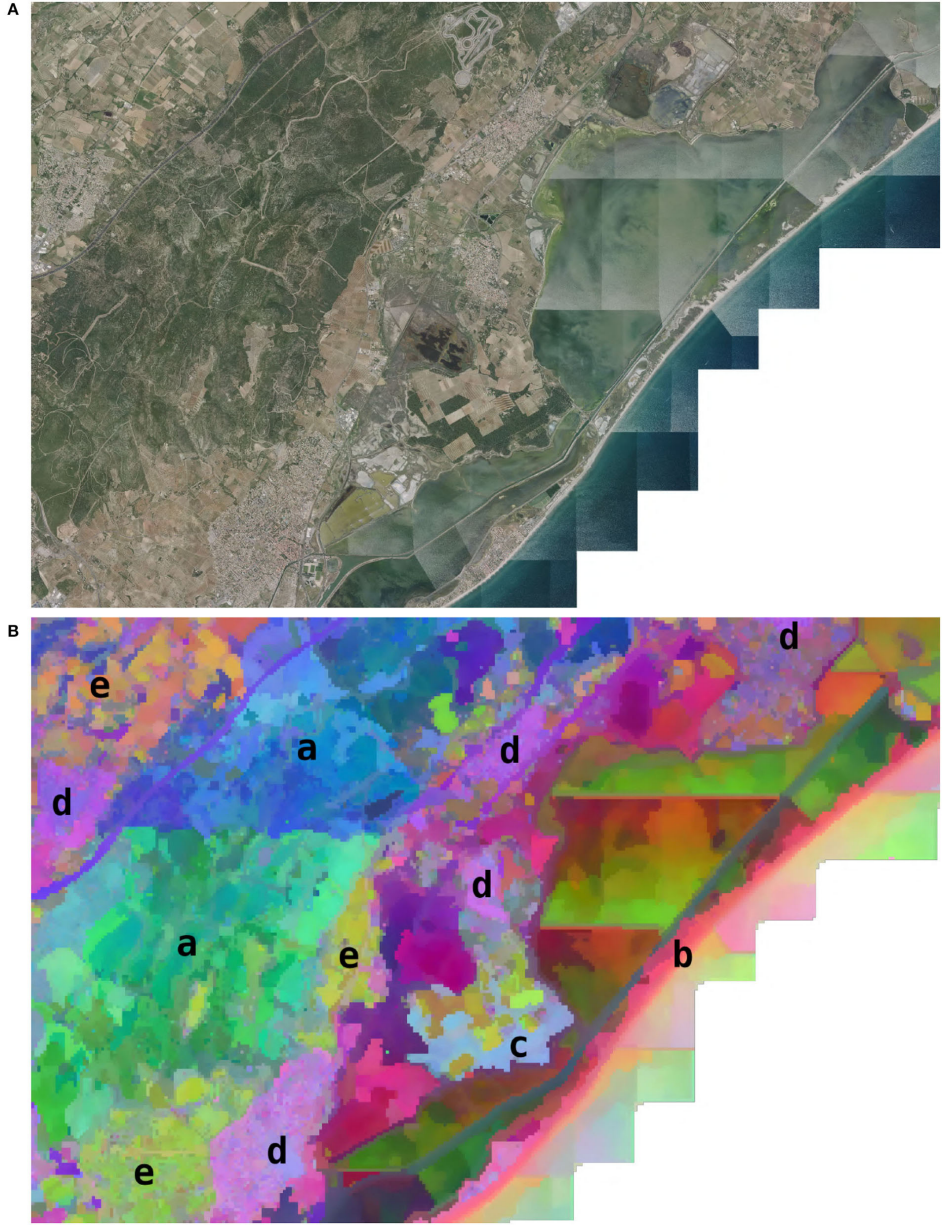
Visualization of the features learned by the CNN in an area of 10 × 15 km on the French Mediterranean coast. **(A)** Remote sensing RGB image. **(B)** Geographical projection (50 m/px) of the t-SNE space (the result of the CNN feature space dimension reduction). Highlighted environments: (a) the mountain of the Gardiole, (b) the beach of the Aresquiers, (c) the Wood of the Aresquiers, (d) villages, and (e) crops.

**Figure 7:**
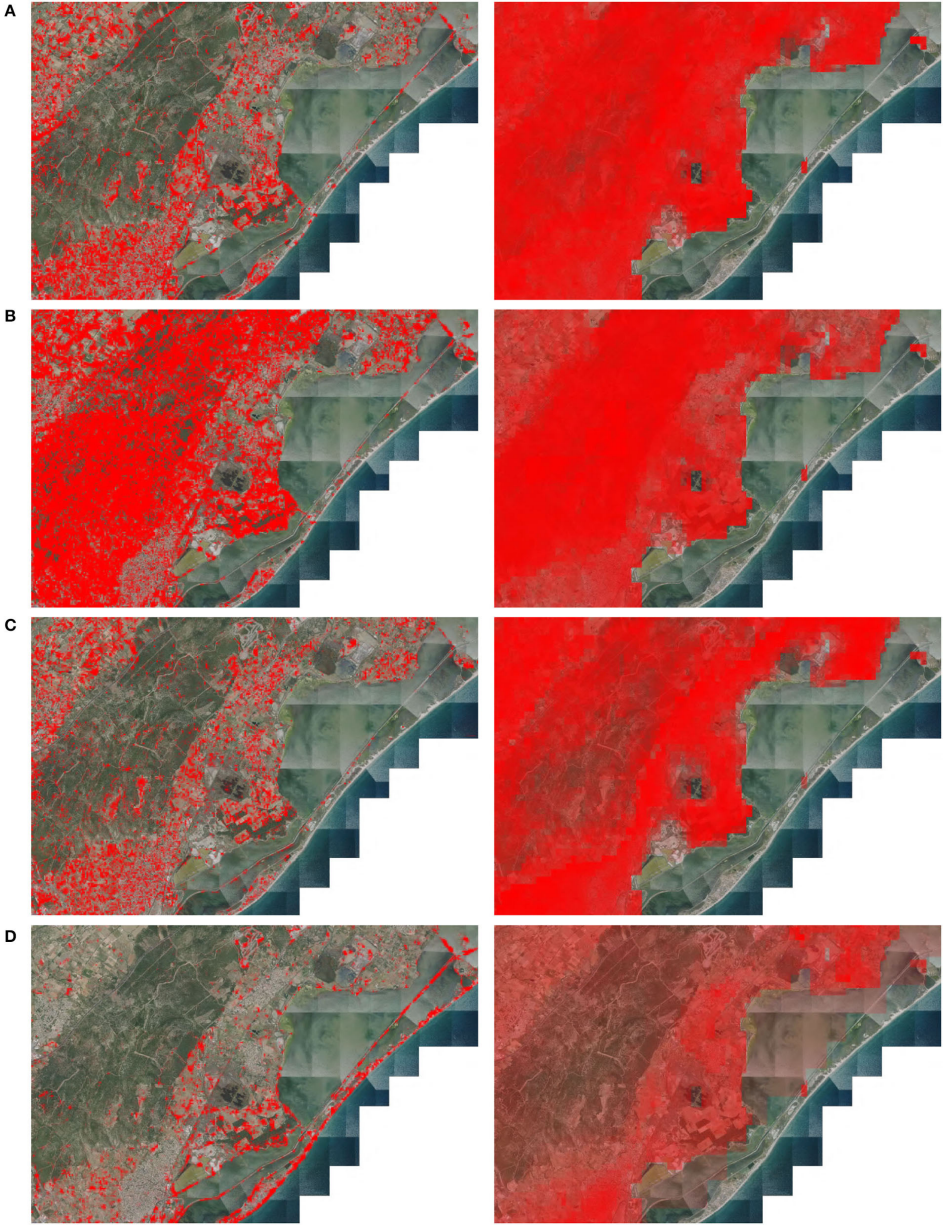
Species CNN logits activation (left) and *MaxEnt* predictions (right) in an area of 10 × 15 km on the French Mediterranean coast (50 m/px). **(A)** *Ailanthus altissima* (Mill.) Swingle. **(B)** *Cistus albidus* L. **(C)** *Capsella bursa-pastoris* (L.) Medik. **(D)** *Anthemis maritima* L.

Figures 6, 7 highlight model ecological significance at a fine spatial scale. Figure 6B is a projection of the 3D t-SNE of the feature space in this area (refer to Section 2.4), it highlights that the model is able to differentiate many fine-scale environments. For example, we can see the temporary ponds (in purplish pink in the center), the highway (purple line in the North), the coast (in light pink), etc. This figure shows good identification of contrasting habitats, in terms of surface and nature. Among the natural habitats of large spatial dimensions, the mountain of the Gardiole (a, natural area of ecological, faunistic, and floristic interest), represented by 2 dominant colors (green in the southwest and blue in the North East), is finely delimited. The southern part of this space presents a gradient of green, from its central plateau located at an altitude of nearly 200 m, to the crops they dominate below (of almond green color). Among the most localized natural habitats, we see that the coastline (b, the beach of the Aresquiers), in light pink color, has indeed been captured over its entire length from the South to the North-East of the figure. Another remarkable, well-identified small-scale habitat is the coastal forest massif dominated by the presence of Pinus (c, the Wood of the Aresquiers), located at the center of the image, in light blue color. The precise delimitation of the outline of this small forest (limited to the south and east by ponds, and to the north and west by crops) shows how well it has been captured, despite its appearance with a fairly strong visual similarity to the forest observed further north in the Gardiole (but presenting a greater specific heterogeneity). The more anthropized habitats are also well-captured since we see that the different villages (d, Frontignan in the South, Gigean in the North West, Vic la Gardiole, and Mireval in the Center, Villeneuve-lès-Maguelone in the North East), whatever their sizes, are well-represented by a unique purplish pink color. The large plots of crops (e) are either represented by a creamy brown color in the North West wine-growing plain, or by an almond-green color for those located between the Gardiole mountain and the sea. Thus, patches of uniform color seem to define a well-defined habitat. This visualization also highlights the impact of data bias. This is particularly visible in the seaside ponds where artifacts coming from the remote sensing data (marked lines due to sun reflections and image reconstruction) seem to create inconsistencies in the feature space. It is difficult to estimate the impact of these artifacts on model learning. Even if they seem to have an important impact on the feature space, they correspond to an area with little or no observation (salt ponds being difficult to access and where only animals can be observed). The model may not have learned to ignore these biases. However, some observed divisions are difficult to explain. In particular, the limits between the green and blue zones seem to be made in the middle of the garrigues without observing any particular bias in the data nor that knowledge of the area seems to explain. This could be the result of multiple factors combined and difficult to untangle such as unidentified bias in data, the influence of near training occurrences, and unidentified environmental shift.

Concerning the maps of logit distributions of the four selected species, the first observation is that the activation is also at fine resolution with notable changes observable from 50 m (distance between points). This confirms that the learning of the model makes it possible to identify a change in the environment on the order of 10 m and that its prediction quickly changes spatially. For the four species, the activation seems to correspond globally to the expected distribution of the species. In comparison, the activations of the *MaxEnt* model, based on much coarser resolution data, do not allow such spatial resolution. With the exception of *Anthemis maritima* L., the dynamics of the *MaxEnt* predictions over the area for the different species also seem to be fairly consistent even if much less fine-grained. We remind for this analysis that only the dynamics of the predictions of the two models (knowing that the species is present in the area but not everywhere) are compared and that we cannot directly compare predictions of the probability of presence. Another point to note is that the environmental rasters are not defined on the seawater ponds present on the map (presence of no data). The *MaxEnt* model does not give predictions in these areas.

*Ailanthus altissima* (Mill.) Swingle., illustrated in Figure 7A, is known to be an invasive tree species of agricultural, urban and forested areas of the Mediterranean and temperate regions, that develop along with transport infrastructures thanks to its winged seeds that are easily spread by the wind. It is well-predicted by the CNN in the various habitats disturbed by human activity. We can note for example the visible activation along the highway (in the northeast) and the activations limited to the agricultural plots, wastelands, and paths in the forest of the massif. We can also see that the model correctly identified the habitats in which this species can not be observed, and which are unfavorable to its development, such as the temporary pools in the center of the map, or the coastline of the Aresquiers. The *MaxEnt* prediction follows roughly this dynamic with the highest values in the areas most impacted by human activity and the lowest values in the ponds (for the few ponds that have data on the rasters). However, the prediction is much less precise and much smoother than for CNN. The *MaxEnt* model tends not to predict on ponds but everywhere else.

*Cistus albidus* L., illustrated in Figure 7B, is a native species of the Mediterranean region and is found in areas of degraded scrublands of Mediterranean forests. Adapted to sunny and dry areas, it can be found over almost all of the territory illustrated in Figure 7B, with the exception of the coast, which explains its broad predicted distribution on the maps produced by the models. It is on the mountain of Gardiole that it is observed in greater numbers, which is well-represented by its strong prediction for this area on both models' maps. For this species, the predictions of the two models are particularly similar. This species is frequent in the region and abundant in the dataset which may allow both models to have a consistent prediction. The main difference still lies in the resolution of the prediction. The CNN prediction can vary quickly where *MaxEnt* is smoother. Unlike the previous species, there are no clearly visible factors to visually explain the rapid variations in predictions by the CNN. It could be due to differences in vegetation type at a fine scale that might be better captured by the CNN-SDM (such as differences in forest cover density).

*Capsella bursa-pastoris* (L.) Medik. Figure 7C is a cosmopolitan herbaceous species observed along paths, in crops lands, and wastelands. Here again, the prediction dynamics of the models are visually quite consistent. Its intense prediction in urban and crops areas is in line with what is known about its ecology. The predictions of the CNN are also finer, it does not predict in the forests, it is noticeable at the level of the woods at the edges of the ponds in the center of the map for example, but predicts the non-forest parcels present on the massif in the middle of the forests.

*Anthemis maritima* L., Figure 7D growing in marine sands, is correctly predicted by the CNN along the coastal line, as well as around the swamps and in the sandy areas between the Gardiole mountain and the sea. However, the *MaxEnt* model seems less consistent for this species. First, as it cannot predict the coastline (due to no data) this highlights a problem for the training of this model for coastal species. Unless the raster coverage is artificially extended to include the entire coastal zone, it is difficult to predict its presence accurately on the coast. Second, apart from the fact that the model is limited in its prediction area, the strongest activations of the prediction are in urban areas, which does not correspond to the ecology of the species. The difficulty in predicting this species by the *MaxEnt* model seems to lie both in the spatial definition of the rasters introducing biases on the observed niche of the species in the training and also in the fact that this species was particularly rare in the dataset (only 24 occurrences). On the contrary, the data used by the CNN and its resolution allow covering completely the coastline and previous studies have already shown the ability of the CNN to predict rare species by joint learning on many species (Deneu et al., 2021). This last result is confirmed in Figure 2. Despite close performances between the CNN and the random forest on the most frequent species, the CNN score is largely superior on the species with few learning occurrences.

We can observe that the activation of logits can change rapidly from one pixel to another (i.e., within 50 m). Contrary to the visualization of the feature space by the t-SNE, here, the activations do not seem to remain uniform on the identified landscape structures. For example, the species *Anthemis maritima* L. is globally predicted along the coastline, but there are some areas of low activation, whereas the t-SNE map seems to be consistent along the entire coastline. This can be the result of several factors. First, the visualization of the feature space by the t-SNE is done using strong dimension reduction, which retains the most important information about the dynamics of the feature space but certainly overlooks weaker variations. The logits are the result of a linear model on the feature space and not of its reduced representation in the t-SNE space. These variations can, therefore, have an impact on the logits without being visible on the map through the t-SNE. Another factor is the learning of the model. The model is evaluated by competing species against each other and the linear models of the last layer producing the activation of the logits are optimized during training to differentiate species which is not equivalent to a prediction of the probability of presence. The model may, therefore, emphasize one species more than another depending on very local factors or sampling bias. One way to limit this effect could be to reduce the size of the representation space (the feature space **z**) leaving less freedom for the model to separate individual species. As the identification of common and representative factors of habitats and communities seems to have more impact, the model would probably tend to favor this aspect.

**Figure 8:**
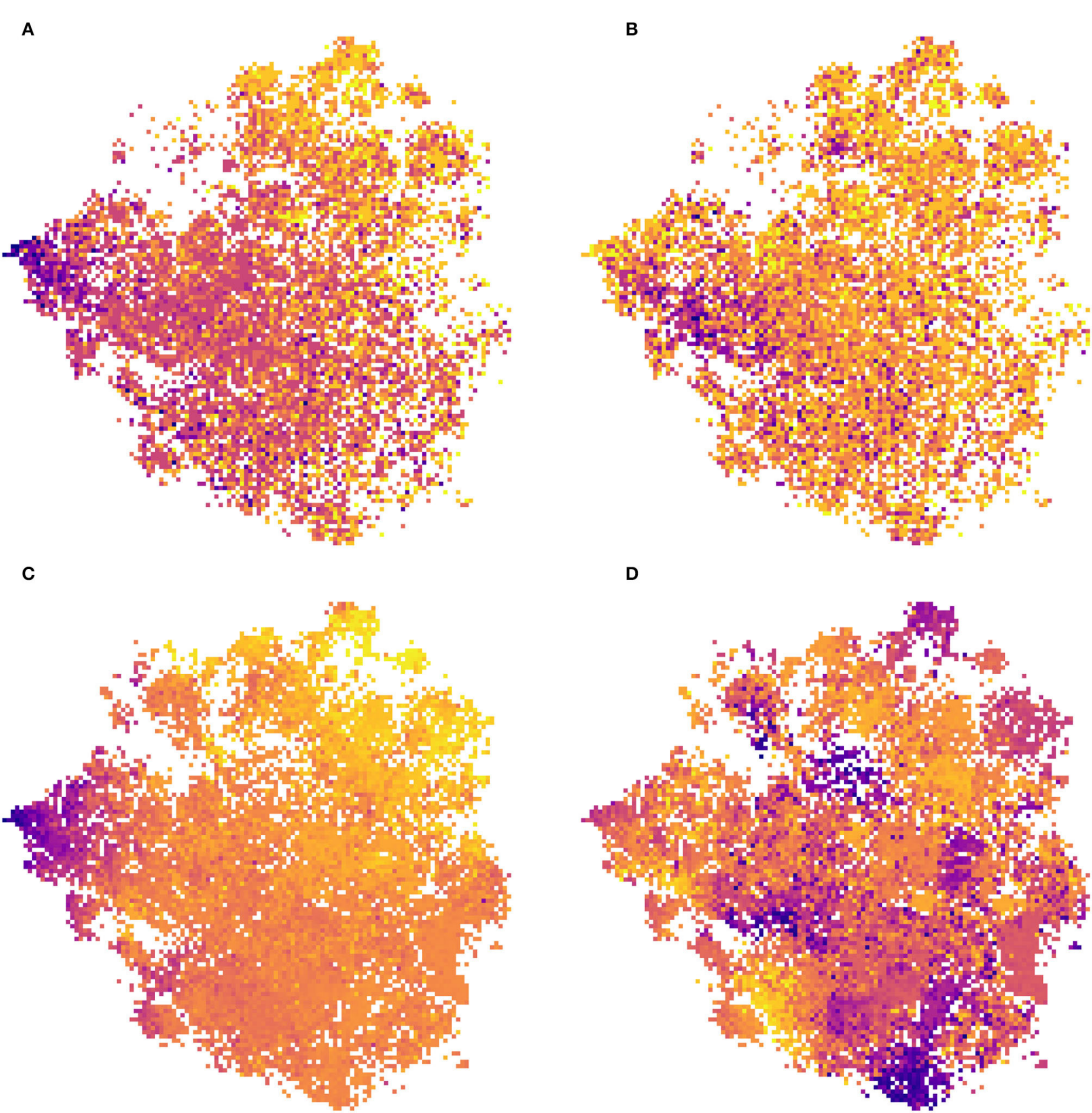
Visualization of two species traits (left) and two environmental variables (right) in the t-SNE space (the result of the CNN feature space dimension reduction): **(A)** species temperature preference (EIV T), **(B)** species light preferences, **(C)** annual mean temperature (bio_1), and **(D)** temperature annual range (bio_7). Artificial colors from purple (lowest values) to yellow (highest values).

Statistical analysis between the environmental or ecological data and the t-SNE space reveals that the CNN captures information strongly related to species ecology.We propose here a very basic analysis of this statistical correlation. We do not directly take into account here the possible collinearities between the different variables, and we use a linear regression whereas the dynamics of the t-SNE space are not necessarily linear (as can be seen in Figure 8D). This may explain why significant relationships are still associated with fairly low coefficients. The idea here is simply to provide a numerical confirmation that the model does capture information directly related to the ecology of the species. Moreover, the significant correlations of this simplistic approach with the highly reduced dimensional space suggest that with further statistical analysis stronger and more precise correlations could be highlighted. Linear models on the t-SNE using species or environmental trait data displays one of the highly significant relationships (Tables 5, 6). For example, the coefficient associated with EIV T (species temperature preferences) is high on both axes of the t-SNE. Looking at the models using Ellenberg traits we see that trait values alone explain a significant portion of the variance in the position of occurrences in the t-SNE space (adjusted R^2^ of 0.111 and 0.231). This highlights that the information captured by the model in the input data is well-correlated with the ecology of the species.

**Table 5:** Ellenberg's species traits linear models on the two axes of the t-SNE space (the result of the CNN feature space dimension reduction).

	**tsne_1**	**tsne_2**
	**Estimate (std. error)**	**Estimate (std. error)**
EIV L	−1.562^***^ (0.236)	2.630^***^ (0.214)
EIV T	−5.035^***^ (0.260)	9.478^***^ (0.235)
EIV K	2.523^***^ (0.389)	0.150 (0.352)
EIV AirH	0.732^*^ (0.378)	−0.869^**^ (0.341)
EIV F	1.461^***^ (0.409)	2.328^***^ (0.370)
EIV R	0.461^**^ (0.213)	−0.686^***^ (0.192)
EIV TroL	2.902^***^ (0.181)	3.851^***^ (0.163)
EIV S	−2.714^***^ (0.366)	−0.981^***^ (0.331)
EIV SoiT	1.649^***^ (0.252)	2.848^***^ (0.228)
EIV N	0.211 (0.212)	−0.598^***^ (0.192)
Intercept	−5.951 (3.947)	−113.831^***^ (3.569)
R^2^	0.111	0.232
Adjusted R^2^	0.111	0.231

* *p < 0.1*;

** *p < 0.05*;

*** *p < 0.01*.

**Table 6:** Environmental linear models on the two axes of the t-SNE space (the result of the CNN feature space dimension reduction).

	**tsne_1**	**tsne_2**
	**Estimate (std. error)**	**Estimate (std. error)**
bio_1	2.837^***^ (0.287)	−0.867^***^ (0.279)
bio_2	−0.774^***^ (0.251)	−1.010^***^ (0.244)
bio_3	3.838^***^ (0.588)	2.667^***^ (0.571)
bio_4	−0.106^***^ (0.008)	0.027^***^ (0.008)
bio_5	0.326^**^ (0.153)	0.116 (0.148)
bio_6	0.225 (0.142)	0.462^***^ (0.138)
bio_8	−0.008 (0.008)	−0.021^***^ (0.008)
bio_9	−0.068^***^ (0.006)	−0.012^*^ (0.006)
bio_10	2.073^***^ (0.415)	0.412 (0.403)
bio_11	−5.772^***^ (0.291)	0.772^***^ (0.282)
bio_12	−0.101^***^ (0.018)	0.005 (0.018)
bio_13	−0.468^***^ (0.064)	−0.144^**^ (0.062)
bio_14	1.303^***^ (0.091)	0.180^**^ (0.089)
bio_15	−0.052 (0.119)	−1.430^***^ (0.115)
bio_16	0.336^***^ (0.035)	0.232^***^ (0.034)
bio_17	−0.220^***^ (0.042)	−0.431^***^ (0.041)
bio_18	0.148^***^ (0.022)	0.047^**^ (0.022)
bio_19	−0.009 (0.022)	0.063^***^ (0.021)
bdticm	0.003^***^ (0.0005)	−0.001 (0.0005)
bldfie	0.042^***^ (0.006)	0.043^***^ (0.006)
cecsol	−0.286^***^ (0.078)	0.422^***^ (0.076)
clyppt	−0.042 (0.344)	−0.286 (0.335)
orcdrc	−0.251^***^ (0.031)	−0.005 (0.030)
phihox	−0.087 (0.068)	1.818^***^ (0.066)
sltppt	0.269 (0.343)	0.133 (0.333)
sndppt	0.215 (0.341)	0.557^*^ (0.331)
Intercept	−81.011^**^ (41.240)	−384.572^***^ (40.078)
R^2^	0.100	0.217
Adjusted R^2^	0.099	0.216

* *p < 0.1*;

** *p < 0.05*;

*** *p < 0.01*.

Figure 8A displays the Ellenberg temperature preference trait (EIV T) and Figure 8C displays the mean annual temperature, both of which are information related to either the species of occurrence or the location of the occurrence, over t-SNE space. One can see in these two representations a strongly pronounced gradient. The coherence between these two figures is expected since the species that have the most affinity with high temperatures are located in the warmest regions and vice versa. If this gradient is so pronounced on the t-SNE space it indicates that information strongly related to the temperature is captured is mostly important in the feature space of the model. Figure 8D represents the Temperature Annual Range of occurrences data on the t-SNE. We can see here that the distribution is characterized by pronounced clusters. Contrary to the annual mean temperature there is no particular gradient on one of the axes but the presence of these clusters also confirms the ability of the model to discern different types of environments. Figure 8B represents the species' light preferences, and here, it is more difficult to see global dynamics. However, a darker cluster (corresponding to species with an affinity for low light) stands out on the left of the image. Figure 3 shows that this area of t-SNE space corresponds to forests. This is another example of information on the ecology of species that the model can capture with the help of VHR remote-sensing data. These results highlight that the information captured by the model is strongly related to the environment and ecology of the species, even though this data was not used directly in model construction. It confirms the potential of remote sensing data for characterizing plant functional types (Ustin and Gamon, 2010; Alleaume et al., 2018).

These results bring some elements for the analysis of the CNN-SDM performances. The simultaneous learning of many species, at a large scale and high spatial resolution, allows the CNN to capture common and consistent information with the ecology of species at several scales ranging from fine landscape to large biogeographic regions.

## 4. Conclusion

In this article, we studied a country-wide species distribution model based on VHR (1m) remote sensing images processed by a convolutional neural network. The evaluation of this model shows that its predictive performance is better than state-of-the-art environmental models while its spatial resolution is several orders of magnitude higher. This strong predictive power at fine scales makes it possible to build maps of potential species distribution at resolutions, spatial scales, and taxonomic scales never before considered. We have illustrated this potential on a few species and a small region in the south of France and compared it with the less fine predictions of a *MaxEnt* model, but it is important to notice that the model has been built on the scale of the whole of France and USA and thousands of plant species. In order to better understand how this model captures ecological information, we have further analyzed the learned features using t-SNE, a powerful dimension reduction technique often used to visualize the representation space of deep learning models. This allowed confirmation that the model captures the relevant landscape and habitat information at fine spatial scales, highlighting the capacity of the model to predict species assemblages locally. In the future study, we plan to combine the remote sensing data with more conventional environmental rasters to further increase the performance of the model. We also plan to extend the approach to the high-resolution mapping of habitats, typically *via* transfer learning approaches that will require little habitat occurrence data.

## Data Availability Statement

Publicly available datasets were analyzed in this study. This data can be found here: https://arxiv.org/pdf/2004.04192.pdf, and the source code of the study can be found here: https://gitlab.inria.fr/bdeneu/high-resolution-cnn-sdm-and-interpretation.

## Author Contributions

BD: conceptualization, data curation, investigation, methodology, software, visualization, writing the original draft, and writing review and editing. AJ: conceptualization, funding acquisition, investigation, methodology, project administration, supervision, validation, writing the original draft, and writing review and editing. PB: conceptualization, funding acquisition, investigation, project administration, supervision, validation, writing the original draft, and writing review and editing. MS: conceptualization, data curation, investigation, methodology, supervision, software, validation, writing the original draft, writing review and editing. FM: conceptualization, investigation, project administration, supervision, validation, writing the original draft, and writing review and editing. All authors contributed to the article and approved the submitted version.

## Funding

This project has received funding from the French National Research Agency under the Investments for the Future Program, referred to as ANR-16-CONV-0004 (#DigitAg) and from the European Union's Horizon 2020 research and innovation program under Grant Agreement No. 863463 (Cos4Cloud project). Models were computed on the Jean Zay super-computer hosted by IDRIS in the context of the French National Grand Equipment (GENCI).

## Conflict of Interest

The authors declare that the research was conducted in the absence of any commercial or financial relationships that could be construed as a potential conflict of interest.

## Publisher's Note

All claims expressed in this article are solely those of the authors and do not necessarily represent those of their affiliated organizations, or those of the publisher, the editors and the reviewers. Any product that may be evaluated in this article, or claim that may be made by its manufacturer, is not guaranteed or endorsed by the publisher.

## Footnotes

^1^National Agriculture Image Program, https://www.fsa.usda.gov

^2^ https://geoservices.ign.fr

^3^ http://osr-cesbio.ups-tlse.fr/~oso/posts/2017-03-30-map-s2-2016/

^4^ https://lpdaac.usgs.gov/products/srtmgl1v003/
